# Functional Cognitive Disorder: Diagnostic Challenges and Future Directions

**DOI:** 10.3390/diagnostics9040131

**Published:** 2019-09-28

**Authors:** Catherine Pennington, Harriet Ball, Marta Swirski

**Affiliations:** 1 ReMemBr Group, University of Bristol, Bristol Brain Centre, Southmead Hospital, Southmead Road, Bristol BS10 5NB, UK; harriet.ball@bristol.ac.uk (H.B.); Marta.swirski@nbt.nhs.uk (M.S.); 2 Centre for Dementia Prevention, University of Edinburgh, 9A Bioquarter, 9 Little France Road, Edinburgh EH16 4UX, UK

**Keywords:** functional cognitive disorder, neurodegeneration, mild cognitive impairment, functional neurological disorder cognition

## Abstract

Functional cognitive disorder describes patients with persistent, troublesome subjective cognitive complaints that are inconsistent with a recognized disease process, and where significant discrepancies are found between subjective and objectively observed cognitive functioning. The etiology is heterogeneous and potentially related to underlying psychological factors. Making a diagnosis of functional cognitive disorder can be challenging and there is the potential for misdiagnosis of early-stage neurodegeneration. We compared neuropsychological findings in three groups: functional cognitive disorder (FCD), mild cognitive impairment (MCI), and healthy controls. Participants were recruited from the ReMemBr Group Clinic, North Bristol NHS Trust, and via Join Dementia Research. Both the FCD and MCI groups showed elevated prospective and retrospective memory symptom scores. Performance on the Montreal cognitive assessment was equivalent in the FCD and MCI groups, both being impaired compared with the controls. The FCD group was younger than those with MCI. We discuss challenges and controversies in the diagnosis of functional cognitive disorder, alongside illustrative cases and proposals for areas of research priority.

## 1. Introduction

Functional cognitive disorder (FCD) describes patients presenting with significant subjective cognitive symptoms that are out of keeping with their observed level of cognitive functioning and not compatible with a recognizable neurodegenerative, psychiatric, or systemic primary cause [1]. In order to make a diagnosis of FCD, a marked discrepancy between self-reported cognitive symptomatology and observed or reported cognitive functioning must be present. This internal inconsistency in symptoms is evident on comparison of symptom severity with performance on neuropsychological testing or everyday “real world’ cognitive ability. For example, above average performance on list learning tasks, or continued work in a skilled profession without difficulty, is inconsistent with a self-report of complete inability to recall any new information over a short time period. The etiology is heterogeneous and likely to be related to underlying psychological factors. FCD is a relatively recently described clinical entity and exists within an expanding spectrum of cognitive diagnoses, which range in severity from subjective cognitive decline (SCD) through to severe dementia. FCD is distinguished from SCD by the presence of significant inconsistency between subjective and objectively observed cognitive functioning, a greater severity of self-reported symptomatology, and resistance to reassurance that observed cognitive functioning is intact. Diagnosing FCD currently relies on expert opinion from a specialist in cognitive disorders. The optimal diagnostic criteria and management strategies for FCD are still areas of debate, and longitudinal studies of prognosis and rate of diagnostic change are lacking. Particular areas of interest are the potential for misdiagnosis of early-stage neurodegeneration, or multifactorial cognitive impairment. The prevalence of FCD is still under investigation and will vary depending on the population being studied. A review of patients attending a tertiary referral cognitive disorders clinic found a third of those aged 60 years or less to have a functional diagnosis [2]. SCD is extremely common, particularly in aging populations. A German LIFE study found that 53% of adults aged 40–79 years reported subjective memory concerns [3], whilst a USA survey identified cognitive concerns in 11.1% of adults over 45 years [4]. The SCD literature has suffered from variable terminology and definitions, which makes estimating true prevalence and incidence difficult.

Some individuals may manifest cognitive symptomatology due to a combination of etiological factors, corresponding to the concept of “functional overlay” seen in systematic and functional neurological conditions, whereby core symptoms caused by an underlying structural disease process are complicated by additional functional features [5]. A dual diagnosis of both epilepsy and psychogenic nonepileptic seizures is not uncommon [6], and individuals with chronic conditions such as multiple sclerosis may manifest additional functional symptoms, which can be misattributed [7]. In the context of cognitive symptoms, there are significant challenges around confirming diagnosis, potential for diagnostic evolution over time, and overlapping conditions. In a clinical setting, rapid access to detailed neuropsychological testing (including effort testing) is often limited, as is the use of CSF biomarkers and advanced imaging techniques, such as amyloid PET. Therefore, the distinction between neurodegenerative and functional or alternative causes for cognitive symptoms frequently depends on a combination of self-report, collateral history, and clinician opinion. The picture is further complicated by memory complaints being a common experience for healthy older adults, and psychiatric symptoms potentially being an early feature of neurodegenerative disease [8]. Therefore, careful exploration of symptom severity, evaluation of discrepancies between self-reported and observed cognitive ability, consideration of potential contributing psychological factors, beliefs or mental health symptoms, and search for evidence of neurodegenerative, toxic, or metabolic causes of cognitive decline are required when considering a diagnosis of FCD. Additionally, a correct diagnosis of FCD does not mean an individual is immune from developing future neurodegeneration.

Here, we discuss issues around prognosis, misdiagnosis, and functional overlay symptoms in FCD. We report data and illustrative cases from a recent study of FCD and MCI patients drawn from a specialist cognitive clinic.

## 2. Methods

Participants with FCD or MCI were recruited from a specialist cognitive disorders clinic (ReMemBr Group, North Bristol Trust). Diagnoses were reached after clinical assessment by a consultant neurologist and clinically appropriate neuroimaging and neuropsychological assessment. The diagnoses of participants recruited from the clinic were reviewed at a multidisciplinary meeting comprising three cognitive neurologists and a consultant neuropsychologist. One patient in the FCD group did not attend for subsequent clinical follow-up, and one MCI participant was recruited from Join Dementia Research. All other FCD and MCI participants attended for additional clinical follow-up in the cognitive disorders clinic.

A diagnosis of FCD was made in patients displaying significant discrepancies between self-reported and observed cognitive functioning (on neuropsychological assessment and/or everyday functioning). Exclusion criteria were evidence of neurodegeneration, toxic or metabolic causes of cognitive decline, major psychiatric disorder, or active systemic disease that could impact cognition.

Diagnoses of MCI were made based on a clinical history of mild cognitive symptoms, consisting of preserved everyday cognitive functioning and mildly impaired performance on neuropsychological testing. Only patients with MCI felt to have a neurodegenerative cause (based on investigation findings and clinical history) were included in the present study. People with toxic or metabolic causes of cognitive decline, major psychiatric disorder, or significant systemic disease that could impact cognition were excluded from this group. One additional participant with MCI was recruited from the Join Dementia Research database; their case notes were reviewed by a cognitive neurologist (C.P.) to confirm the diagnosis. Healthy controls (HC) were recruited from a local database of adult research volunteers and the Join Dementia Research database. Controls self-identified as having no significant cognitive complaints. We did not exclude persons with mild mood or anxiety symptoms that (in the opinion of the cognitive neurology team) would not be expected to impact cognition.

Participants underwent neuropsychological testing, and demographic data were collected. This included the Montreal Cognitive Assessment (MoCA) [9], the Test of Premorbid Functioning—UK version (ToPF) [10], and the Prospective and Retrospective Memory Questionnaire (PRMQ) [11]. The MoCA is a 30-point brief cognitive assessment, with one further bonus point awarded to adults with formal education of 12 years or less. The PRMQ is a brief questionnaire asking respondents to answer 16 questions about minor everyday memory errors, using a 5-point Likert scale (from “never” to “very often”). Half of the questions refer to prospective memory, and half to retrospective. The responses are assigned values from 5 for “very often” to 1 for “never” and summed, and total scores are a minimum of 16 and maximum of 80. The ToPF provides an estimate of premorbid IQ, based on knowledge of the pronunciation of irregularly spelt words. Subjects are asked to read words with irregular grapheme–phoneme association aloud from a stimulus card. The words increase in difficulty, and the task is finished and a score assigned when errors in pronunciation occur.

The project was given Research Ethics Committee approval by the South West—Cornwall and Plymouth Research Ethics Committee, REC reference 15/SW/0298 and IRAS project ID: 188539. All participants provided informed consent. The study was funded by the BRACE charity.

Statistical analysis was undertaken using IBM SPSS Statistics version 22 and Stata version 15.1. Group comparisons were undertaken using 1-way ANOVA, or alternative tests where data did not meet assumptions for parametric data.

## 3. Results

A total of 21 people with FCD, 17 with MCI, and 25 HC participated in the main study. In the FCD group, all but two participants had structural neuroimaging (CT, MRI, or both). In the MCI group, all the participants had structural imaging, and six had additional functional imaging (HMPAO SPECT or DAT). The group demographics are shown in Table 1, and analysis of group differences on the PRMQ are shown in Table 2.

### 3.1. Case Studies

An additional two patients were recruited into the study and completed informed consent and study procedures. A subsequent case review was performed on study completion, and diagnostic uncertainty was raised in both cases. They are presented separately from the main study cohort, to illustrate potential diagnostic difficulties and change in the cognitive clinic population.

#### 3.1.1. Case 1

A person in their sixties presented following several years of behavioral and cognitive changes. Symptom onset began around the time of a job change and an uncomplicated elective operation with subsequent opiate prescription. There was no evidence of excessive or dependent opiate use, and cognitive symptoms did not remit on the gradual cessation of opiates. The individual was less able to manage a complex occupation and had reduced empathy and extraversion. Episodic word finding difficulties were reported, exacerbated by fatigue. Symptoms improved during holiday periods.

Examination was notable for mild extrapyramidal signs. HMPAO SPECT showed frontal hypoperfusion. Negative investigations included blood tests for genetic and autoimmune causes of neurodegeneration.

On neuropsychological testing, the participant passed tests of performance validity. Most scores were in the average range, and a minority of subsets were in the low-average range. On subjective questionnaires, memory was self-rated as good, whereas their spouse rated it as significantly impaired. Depression, anxiety, and stress self-report scores were in the average range. An initial diagnosis of MCI was made.

**Figure 1:**
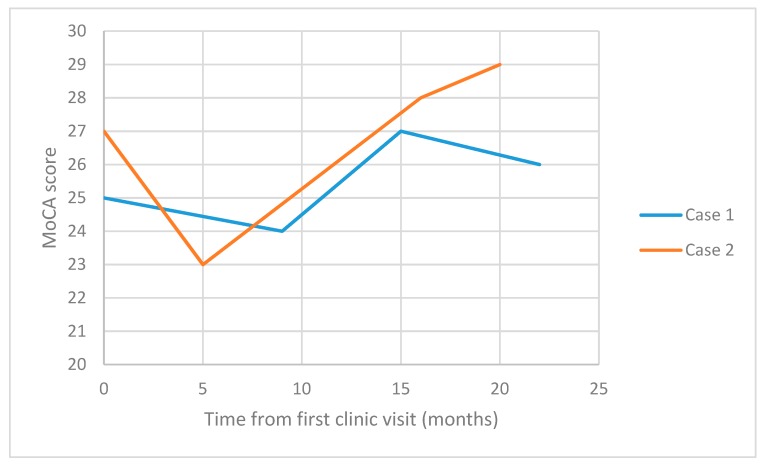
Serial Montreal Cognitive Assessment (MoCA) scores (bonus point included if appropriate) for illustrative cases one and two.

On follow-up, MoCA scores showed an improving pattern (Figure 1). Neuropsychological testing was repeated after a one-year interval, and during this assessment, anxiety was evident. The patient also showed catastrophic interpretation of normal memory lapses. Cognitive performance results were stable or improved. A neuropsychiatric opinion highlighted exaggerated interpretation of normal memory lapses, and a revised diagnosis was made of SCD, and possible functional overlay. The individual remains under follow-up, and the trajectory of improved neuropsychology now points away from neurodegenerative processes.

#### 3.1.2. Case 2

A person in their fifties was referred for investigation of cognitive symptoms and extensive vascular change on MRI of the brain. Two years earlier, the individual had experienced a critical illness requiring ITU stay. Following this, they had initially been able to return to previous employment but reported cognitive slowing, word-finding difficulties, and problems remembering appointments. These symptoms and stress resulted in a subsequent decision to cease work. The individual reported lack of motivation, depression and anxiety, and progressive cognitive symptoms. Their partner felt that the cognitive symptoms had been static since ceasing work. The clinical picture was complicated by poor sleep, chronic pain and opiate use, and alcohol intake. They were a current cigarette smoker, had a family history of cardiovascular disease in a first-degree relative, and dementia in second-degree relatives.

Neurological examination demonstrated jerky saccadic movements, limb weakness related to previous surgery, and chronic pain. There were no pyramidal or extrapyramidal signs. A brain MRI showed small vessel ischemic change out of proportion to age, which showed mild progression on follow-up imaging a year later. Blood tests for vasculitis and inflammatory conditions (including CADASIL genetics) were unrevealing.

On neuropsychological testing, they passed tests of performance validity. Scores were low average or borderline impaired on several subsets (in particular, on word list recall, alongside average performance on short story recall). Depression and anxiety screening questionnaire scores were elevated. There were inconsistencies between self-reports of progressive cognitive symptoms in the absence of evidence of progressive cognitive decline on clinical review, and the collateral history of cognitive stability. Significant contributing adverse psychological factors were present that were felt to be impacting cognition, in conjunction with toxic and metabolic factors and vascular disease. A diagnosis of multifactorial MCI was ultimately made, and management included optimizing vascular risk factors, referral for psychological therapies, and support to attempt to reduce opiate use.

## 4. Discussion

In this case series of adults with FCD and neurodegenerative MCI, we found those with FCD to report an equivalent burden of cognitive symptomatology to people with MCI. Total PRMQ T scores were 33.3 in the FCD group and 35.1 in the MCI group, approximately 1.6 SD below the normative population mean [11]. This finding is in line with previous literature on functional neurological disorder, where self-ratings of disability by patients with functional symptoms are as severe or worse than those with organic neurological disease [12]. Performance on the MoCA was also indistinguishable between FCD and MCI, as both groups showed significantly impaired scores compared with the controls.

The FCD group showed lower performance on the ToPF, compared with the controls and the MCI group. The ToPF is designed to estimate premorbid cognitive abilities but is known to potentially be vulnerable to the impact of neurodegeneration and nonorganic underperformance [13]. Martin et al. examined links between performance validity test results and the ToPF, finding those failing validity tests were more likely to have a lower ToPF score than demographically predicted. People with FCD frequently show an invalid pattern of performance on neuropsychological testing [2], and it is possible that the ToPF results in the present FCD group may not accurately reflect their true premorbid baseline.

Limitations of this study include a lack of “gold standard” diagnostic criteria for FCD. Diagnostic criteria are still in evolution, and clinician judgement remains the mainstay of diagnosis. The ReMemBr Group cognitive clinic receives complex referrals from primary and secondary care; therefore, there is likely to be referral bias towards less straightforward diagnostic scenarios, which may have influenced the nature of the participants recruited in the present study. A common problem across cognitive research is a tendency for research participants to be of above population norm levels of education and socioeconomic status. Therefore, we would not consider our study sample to be truly representative of the local population, and it is possible that significant cultural differences may impact how FCD manifests and is diagnosed. Larger studies of more diverse populations in different world regions are needed to further our understanding of FCD.

Cognitive disorders are traditionally thought to reflect a binary, unidirectional process. They are present or absent, progress along a trajectory of increasing severity, and with the exception of rare, treatable causes of cognitive decline, they do not remit. This very linear view of cognition is increasingly at odds with evidence from large studies of populations with MCI, where a significant percentage of affected individuals revert to normal or near-normal cognition over time [14]. Those who improve retain an increased future risk of MCI or dementia compared to those who have not previously been diagnosed with MCI, and thus, they may move between diagnostic categories more than once during their cognitive journey [15,16]. Individuals with SCD are also highly heterogeneous. The SCIENCe cohort study subdivided people with SCD into those with preclinical Alzheimer’s disease (AD; based on CSF or PET amyloid positivity), those with very mild psychiatric symptoms, and the remainder of individuals with features of neither [17]. A classification of preclinical AD was associated with older age and Apolipoprotein E4 status. Those with psychiatric features reported a greater degree of cognitive symptomatology than those with preclinical AD. Other studies of the long-term prognosis of SCD have identified a variety of potential clinical trajectories, including symptom remittance. Those with persistent symptoms reported over a number of time points appear to be at greater risk of progression to MCI or dementia, whilst those who intermittently report cognitive symptoms are not at increased risk, compared to people who have never reported cognitive symptoms [18]. Clearly, in the instances of SCD and MCI, diagnostic status is often a dynamic concept.

Our case reports highlight the potential for cognitive multimorbidity and diagnostic change over time. Case 1 was initially diagnosed with MCI, but this was revised on clinical follow up to SCD, with a possible element of functional overlay. Potential “red flags” for future cognitive decline are present in this case, based on the abnormal HMPAO SPECT, and cognitive changes being noted by close informants. Symptoms reported by significant others is regarded as a pointer towards neurodegeneration, as opposed to self-reported symptoms [19]. Contrarily, the improvement in MoCA performance would go against a neurodegenerative process, and the observed tendency to catastrophic thinking is a positive feature of functional symptoms. Given the diagnostic ambiguity, it could be argued that this patient might benefit from advanced neurodegenerative biomarker analysis with amyloid or tau imaging, and CSF analysis. However, these tests are not yet widely available in the UK NHS, and there is potential for iatrogenic harm by over-investigation, either through physical complications or adverse psychological consequences.

The diagnosis of FCD depends on expert clinician opinion, with the use of targeted clinical investigations. The clinical picture in FCD is of a significant burden of persistent cognitive symptoms, which cause patient distress and worry, but objective cognitive deficits are inconsistent with self-reported symptoms—this may be based on neuropsychological assessment, or self or collateral reports of activities requiring a high level of cognitive functioning. The discrepancy between symptoms and behavior is key to the diagnosis of FCD, and additional positive features are resistance to reassurance, repeated medical consultations, and failure of performance validity tests.

Case 2 illustrates the multifactorial nature of cognitive changes experienced by many patients, and the potential for functional symptoms to overlay other cognitive pathologies. The patient had been exposed to significant serious systemic morbidity, had evidence of neuronal injury on neuroimaging, in addition to ongoing issues with psychoactive medications, alcohol, and sleep pathology. There were also significant mental health symptoms, and the clinical impression was of functional symptoms contributing to the cognitive picture. It is extremely challenging to unpick the contributions of these different factors. A pragmatic management strategy should aim to improve potentially reversible factors, such as sleep, mental health, and cognitive toxin intake. Longitudinal follow-up is often needed, as the pattern of cognitive change (or lack thereof) is of diagnostic value, particularly to avoid missing neurodegeneration in complex individuals. The possibility of mental health symptoms arising as part of a dementia prodrome should be considered, particularly in older adults and those without a past history of mental health disorders.

Findings from our case series comparison of groups with FCD, MCI, and healthy controls show that PRMQ scores were equivalent in both the FCD and MCI groups, as was MoCA performance. This is despite expert opinion that those in the FCD group reported cognitive symptoms that were disproportionate to their observed cognitive functioning. Therefore, the PRMQ may not capture sufficiently detailed self-reported symptoms to be used diagnostically. The presence of a major discrepancy between subjective and objective cognition remains the mainstay of FCD diagnosis. More detailed exploration of neuropsychological features of FCD is warranted to seek out potential condition specific features that can aid in diagnosis. The potential for a diagnosis of FCD to change over time has not yet been explored, and there are little data available on the long-term prognosis. The optimal extent of investigations and follow-up is also unresolved. A deep phenotyping study of FCD will shortly open at the University of Edinburgh [20]. It is hoped that this and other future studies will establish clear diagnostic criteria for FCD and provide insight into causative mechanisms and avenues for potential therapeutic developments. Clinicians and researchers should be mindful of the potential for both diagnostic overlap and diagnostic change over time.

## Figures and Tables

**Table 1:** Group demographics and cognitive functioning (mean values; significant *p* values in bold).

	FCD	MCI	HC	FCD vs. HC *p* Value	FCD vs. MCI *p* Value	MCI vs. HC *p* Value
Female:Male	10:11	8:9	18:7		0.15*	
Age (years)	58.3	72.1	60.8	0.30	**<0.01**	**<0.01**
Years of Education	13.8	14.4	14.7	0.61 *		
MoCA	23.9	23.3	27.8	**<0.01**	0.52	**<0.01**
ToPF	46.5	55.1	57.2	**<0.01**	**0.04**	0.87

* No significant difference across samples found; therefore, multiple comparisons not performed. Gender: Pearson’s Chi-square = 3.7, *p* = 0.15; Age: Kruskal-Wallis test. Chi-square = 12.7, df = 2, *p* < 0.01; Years of education: one-way ANOVA. F = 0.5, df = 60, 2, *p* = 0.61; MoCA: Kruskal–Wallis test. Chi-square = 19.0, df = 2, *p* < 0.01; ToPF: Kruskal–Wallis test. Chi-square = 7.6, df =2, *p* = 0.02.

**Table 2:** Mean scores on the Prospective and Retrospective Memory Questionnaire.

	FCD	MCI	HC	FCD vs. HC *p* Value	FCD vs. MCI *p* Value	MCI vs. HC *p* Value
Prospective memory (raw score)	29.2	27.6	18.6	**<0.01**	0.72	**<0.01**
Retrospective memory (raw score)	25.2	25.4	16.4	**<0.01**	1.00	**<0.01**
Total T score (Prospective + Retrospective)	33.3	35.1	54.7			

Higher raw scores indicate greater self-reported memory symptoms. Lower T scores indicate greater symptomatology. T scores have a mean of 50 and SD of 10; therefore, both FCD and MCI were both approximately 2 SD below population norms. T scores were obtained using tables in Crawford et al. 2003 (Crawford et al., 2003). Analyses were repeated as an ANCOVA (controlling for age), obtaining consistent results. One-way ANOVA with Tukey post-hoc comparisons. Prospective memory raw score, F (2,60) = 19.91, *p* < 0.01. Effect size η^2^ 0.40 Retrospective memory raw score, F (2,60) =16.5, *p* < 0.01. Effect size η^2^ 0.35.
